# Unsteady thermal Maxwell power law nanofluid flow subject to forced thermal Marangoni Convection

**DOI:** 10.1038/s41598-021-86865-0

**Published:** 2021-04-06

**Authors:** Muhammad Jawad, Anwar Saeed, Taza Gul, Zahir Shah, Poom Kumam

**Affiliations:** 1 Department of Mathematics, Abdul Wali Khan University, Mardan, 23200 Khyber Pakhtunkhwa Pakistan; 2 Department of Mathematics, City University of Science and Information Technology, Peshawar, 25000 Khyber Pakhtunkhwa Pakistan; 3 Department of Mathematics, University of Lakki Marwat, Lakki Marwat, 28420 Khyber Pakhtunkhwa Pakistan; 4 Fixed Point Research Laboratory, Fixed Point Theory and Applications Research Group, Center of Excellence in Theoretical and Computational Science (TaCS-CoE), Faculty of Science, King Mongkut’s University of Technology Thonburi (KMUTT), 126 Pracha Uthit Rd., Bang Mod, Thung Khru, Bangkok, 10140 Thailand; 5 Department of Medical Research, China Medical University Hospital, China Medical University, Taichung, 40402 Taiwan

**Keywords:** Engineering, Mathematics and computing, Physics

## Abstract

In the current work, the unsteady thermal flow of Maxwell power-law nanofluid with Welan gum solution on a stretching surface has been considered. The flow is also exposed to Joule heating and magnetic effects. The Marangoni convection equation is also proposed for current investigation in light of the constitutive equations for the Maxwell power law model. For non-dimensionalization, a group of similar variables has been employed to obtain a set of ordinary differential equations. This set of dimensionless equations is then solved with the help of the homotopy analysis method (HAM). It has been established in this work that, the effects of momentum relaxation time upon the thickness of the film is quite obvious in comparison to heat relaxation time. It is also noticed in this work that improvement in the Marangoni convection process leads to a decline in the thickness of the fluid’s film.

## Introduction

In the last two decades “Nanofluid” attracts the attention of researchers because of its high thermal conductivity and novel applications in different branches of science, engineering, and technology. The conventional liquids consume low thermal conductivity and thus they become inadequate for several heat transfer issues. The study of nanofluids is very important for the reason of its unique application that enhances the transfer of heat. That’s why scientists take an interest to use nanofluid instead of regular fluids. Many researchers are interested to study nanofluids because of their higher thermal capability. Thermal conductivity is a unique characteristic of nanofluid which is much higher than any other fluid. A conventional fluid can be transformed into a nanofluid by suspending nano-sized particles also termed as nanoparticles into that fluid. The early studies by Choi^1^ concerned mixtures of common fluids and nano-sized solid particles. These fluids were named as naofluids. After Choi’s work, many researchers diverted their attention to discussing the nanofluid characteristics focusing upon the heat transfer capabilities of this class fluid. Aziz et al.^2^ have explored the production of irreversibility in MHD Maxwell nanofluid flowing upon stretching surface under the influence of varying thermal conductivity. The authors of this work have also exposed the flow system to slip condition and external heat source. Currently, Gul and Ferdous^3^ have carried out an experimental examination to study the stable distribution of graphene nanoparticles between two rotating disks. The authors of this work have transformed the model equations to dimensionless form by employing a suitable set of transformations and then have solved that set numerically. Maxwell nanofluid flow past an elongating sheet was examined by Sameh et al.^4^. In this study, they used porous surface along with nonlinear thermal radiations and heat generation/absorption.

The flow and thermal transmission in a limited fluid film over a constant exterior are significant in physical importance. The MHD of a limited liquid medium, for example, a thin fluid film, on an extending slip was first measured by Wang^5^ who by methods of similarity alteration diminished the equations to non-linear ODEs. The impacts of viscid scattering and inner warmth generation on stream and warmth move in a thin film on an uneven extending slip and it ought to be noticed that an overall external heat was contemplated by Aziz et al.^6^. The temperature and velocity profiles were tackled utilizing the Homotopy Analysis Method. The temperature-dependent constraint expands; the dimensionless heat heightens while the warmth transmission rate diminishes. Expanding the power indices has been appeared to have the effect of diminishing the heat of thin-film flow. Crane^7^ explained the consistent two-dimensional progression of a Newtonian liquid, the prior investigations of the fluid movement brought about by the extending elastic level sheet.

The heat transfer of power law liquid past an extending slip with the influence of Marangoni convection was conceded out by Liu et al.^8^. However, as for the polymer solutions with both viscoelasticity and shear thinning properties, it is rarely involved in the work of heat transfer employing the fickle thermal conductivity into Cattaneo–Christov heat flux theory. Resulting in this pioneering work, Usha and Sridharan^9^ protracted the issue to the axisymmetric formation for a plate with an uneven extending indication utilizing a similar outward velocity. A steady solution with finite thickness and the reverse stream has been originated in the steady case $S = 0.0$. Moreover, no solutions are established for $S > 4.0$ which just implies that no similarity solutions occur and does not relate to non-similar solutions. The investigation for unsteady nanofluid film brought about by a linear extending velocity over a level flexible slip by utilizing the scientific nanofluid model was depicted by Tiwari and Das^10^. The nanoparticles when inundated in a liquid are fit for expanding the warmth move limit of the base liquid. The conveyance equations are illuminated statistically with a limited volume style utilizing the SIMPLE algorithm. As a solid volume fraction expands, the impact is increasingly articulated. Nandeppanavar et al.^11^ examined the warm dissemination in Newtonian liquid film stream over a flat slip whose extending amount and heat were elements of both space and time. Nasir^12^ mathematically investigated a coupled mass and heat impacts for Newtonian fluid film buildup. The expansion of the porosity improves the water film buildup. An expansion in permeable layer thickness upgrades the water film buildup. A lessening of the porosity and of the permeable layer thickness upgrades the warmth and mass exchange exhibitions over the fluid-vapor interface. The diminishing of the cooling heat motion or the expansion of the inlet gas and fluid temperature lessens the water film buildup.

Numerous researchers have done lots of studies and made extraordinary accomplishments in power-law liquids. The film movement on an uneven extending superficial for a power-law liquid was contemplated by Andersson et al.^13^. For power law liquids, the novelists establish a similarity alteration to lessen the overseeing equations to a similarity equation and comprehended it statistically. Ahmed et al.^14^ investigated Stream and heat transference examination of an electrifying directing MHD power-law nanofluid is helped out over the circular part conduit, affected by the consistent pressure gradient. With the expansion in attractive field parameter and volume fraction of nanoparticles improvement in heat transmission amount has been seen in both shear thinning and shear thickening nanofluids. Improvement in the warmth move rate increments on account of shear thickening nanofluid by expanding the estimation of the attractive field parameter. The effect of a higher attractive field parameter gets articulated and, causes a lessening in the impact of volume division of nanoparticles on account of shear thickening nanofluid, which has been seen from the velocity distributions. The flow problem for a power-law fluid film over an uneven extending outward utilizing the homotopy analysis method was carried out by Wang and Pop^15^. Si et al.^16^ explored the heat transferal wonder of a power-law fluid laminar film joined by power-law warm conductivity. The warm conductivity is thought to be a power-law reliant on the velocity gradient. Aziz et al.^17^ investigated the impact on a power-law liquid former a permeable level disk installed in the Darcy type permeable medium. The subsequent arrangement of ordinary differential equations is settled numerically utilizing Matlab bvp4c Solver. Silva et al.^18^ introduced a different scientific model almost a power-law liquid streaming in a passage partly loaded up with an isotropic and homogeneous permeable medium, and acquired mathematical outcomes. Ahmed et al.^19^ further reached out to the unsteady limit layer stream and warmth move of intensity law liquid model over a radially extending sheet, and another non-linear dispersion model regarding the laminar limit level movement of power-law liquid was presented by Lin et al.^20^. Hainke et al.^21^ examined an endeavor was made to quickly sum up the current status of the utilization of attractive fields in crystal growth development and under microgravity conditions. In this manner, the primary accentuation was put on the Research and development commitments acquired by the Crystal growth development Lab in these fields.

Witkowski and Walker^22^ deliberated the axisymmetric stream determined by Marangoni convection and turning attractive field in alignment for altered Marangoni number and short Prandtl number estimations. A correspondence examination for simply the velocity distribution for the Marangoni stream that is fundamentally the same as this deduction yet the outcomes are successfully restricted to superficial tension varieties that are linear recognized with the superficial point were introduced by Arafune and Hirata^23^. A portion of the papers generally pertinent to this effort incorporate the significant level examination of the Marangoni stream assumed by Okano et al.^24^ that provided the overall patterns for the variety of the Grashof number with the Reynolds, Prandtl, and Marangoni numbers. Hirata and his collaborators empirically and statistically explored the Marangoni stream for different elements in geometries with level exteriors applicable to this effort. The impacts of solutal Marangoni convection on flow and mass transport marvels are talked about^25^. Chen^26^ stretched out the Newtonian liquid to the power-law liquid and analyzed the constrained thermal Marangoni convection flow highlight of the thin film. The thermo-capillary force will in general condense the film and marks in a nearby least of velocity, a more extensive Thermo-capillarity shows an increasingly articulated impact on the temperature and velocity profiles at a lesser changed Prandtl number.

Maxwell fluid is one of the non-Newtonian fluids and it was originally modeled by Maxwell to obtain viscoelastic performance of air through the dynamic theory of gasses. In physical situations, many fluids, such as glycerine, crude oils, Eos, blood and some other polymeric materials, behave like Maxwell fluids. Christov^27^ thought the simplification of Fourier's law identified as the Maxwell–Cattaneo law, has been reevaluated from the perspective of substantial invariance. Lin et al.^28^ investigated the Attractive field impacts in the power-law limited thin film over an uneven extending slip with varying warm conductivity was concentrated. The governing PDEs were changed into an arrangement of strong non-linear ODEs utilizing a comparison alteration. These ODEs were comprehended statistically utilizing the package BVP4C. They deliberated that the attractive field will in general log jam the speed and to expand the heat of the liquids. The impact of thermocapillarity on the stream and warmth move in a thin fluid film on a flat extending slip was investigated by Dandapat et al.^29^. The administering limit layer conditions for thermal energy and momentum are decreased to a lot of combined ODEs by using an exact similarity transformation. The uneven movement and warmth transference of power-law nanoliquid thin film over an extending slip with varying attractive field and power-law velocity slip impacts was investigated by Zhang et al.^30^.

The shear thinning effect is more necessary for the viscoelastic fluids like Maxwell fluid to reduce their viscosity to gain the desirable outputs for the various engineering phenomena’s like hydraulics calculations and decreasing in the pumping pressures. Furthermore, highly shear thinning fluids (power law model fluids) improve the flow efficiency of the viscoelastic fluids. Therefore, we have selected the current model using the combination of Power law model with Maxwell fluid^31^. Zhang et al.^32^ have discussed Maxwell-power-law constitutive equation, which can simultaneously describe both shear thinning and viscoelasticity, is established based on a rheological experiment with welan gum solution. Few related work to our research work are found in^33–37^. Liang et al.^38^ have investigated the uni-dimensional exact for nonlinear Schrodinger problem with a description of dynamics Einstein solution. Wang et al.^39^ have discussed the transportation characteristics for F = 2 spinor one dimensional BEC loaded optical lattice in light of five components semi discrete GP equations. Ji et al.^40^ have discussed the dynamical creation for fractional half quantum vortices Einstein condensate of sodium atoms. Wang et al.^41,42^ have carried out further investigation about the related concept.

In light of the previously mentioned examinations, in the present investigation, we considered the power-law nanofluid thin film flow over an extending surface with the magnetic effects and Joule heating impacts. The Maxwell-power-law liquid flow and heat transfer possessions with variable thickness film are also examined. The Maxwell and Power law model fluids are considered in a single constitute equation and this is the first attempt to consider these two fluids in combined. Thermal Marangoni convection; MHD and solid nanoparticles are also used in the form of extension in the existing literature.

It is applicable in polyamide (PA) barrier films, plastic packaging and new energy etc. In the part of mathematical formulation, our model is formulated by using appropriate similarity transformation and then has solved the nonlinear system by HAM.

## Basic equations of Maxwell and power law model

Since for Maxwell fluid we have^27^

1 $$ \left( {1 + \lambda \frac{D}{Dt}} \right){\varvec{S}} = \mu_{1} {\varvec{A}}. $$

In Eq. (1) $\lambda$ is relaxation time, $\mu_{1}$ is dynamic viscosity*,* ${\varvec{A}}$ is Rivlin–Ericksen tensor while ${\varvec{S}}$ is extra stress tensor. Further we have $\mu_{1} = \mu \dot{\gamma }^{n - 1}$ where $\dot{\gamma }$ signifies the shear rate, $n$ symbolizes a power-law index. The Maxwell power law equation cam be described as

2 $$ \left( {1 + \lambda \frac{\partial }{\partial t}} \right){\varvec{S}} = \mu \dot{\gamma }^{n} {\varvec{A}}. $$

For one-dimensional shear flow, a Maxwell-power-law fluid constitutive equation is simplified as: where *τ* represents the shear stress.

3 $$ \left( {1 + \lambda \frac{\partial }{\partial t}} \right)\tau = \mu \dot{\gamma }^{n} . $$

## Problem formulation

**Figure 1:**
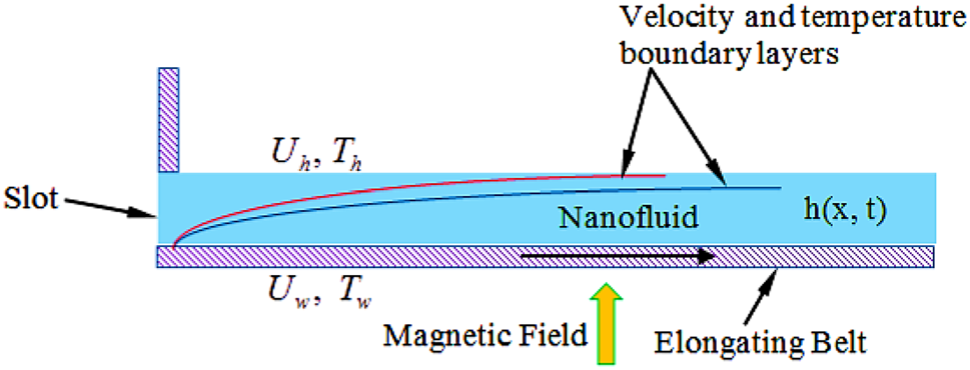
Geometry representation of the flow.

We explore the unsteady 2D flow of an incompressible Maxwell-power-law nanofluid for a finite film past an extending surface. The outcome of heat transfer and the consistent magnetic field $\beta_{0}$ is deliberated in the flow. The temperature and flow filed are described respectively as $T,\,\,\left( {u,\,\,v,\,\,0} \right)$. For current flow problem $x{\text{ - axis}}$ considered along stretching sheet while $y{\text{-axis}}$ is normal to it (see Fig. 1).

The stretched sheet through $\vec{U}_{w} \left( {x,t} \right) = \frac{bx}{{1 - \alpha t}},$ here $b$ and $\alpha$ signifies widening constraints and y-axis is perpendicular to it. From Eq. (2), the continuity and the momentum equations are^32^:

4 $$ u_{x} - v_{y} = 0, $$

5 $$ u_{t} + uu_{x} + vu_{y} + \lambda \left( {u^{2} u_{xx} + 2uvu_{xy} + v^{2} u_{yy} } \right) + \frac{{\sigma_{nf} \beta_{0}^{2} }}{{\rho_{nf} }}u = \frac{{\mu_{nf} }}{{\rho_{nf} }}\left( {\left| {u_{y} } \right|^{n - 1} u_{y} } \right)_{y} . $$

Moreover, the stretching sheet temperature takes as $T_{w} = T_{0} + T_{ref} dbx^{{\frac{3n + 1}{{n + 1}}}} \left( {1 - \alpha t} \right)^{{ - \frac{3n}{{n + 1}}}}$, $T_{0}$*.* So energy can be articulated as^30^

6 $$ T_{t} + uT_{x} + vT_{y} + \frac{{\sigma_{nf} \beta_{0}^{2} }}{{\left( {\rho_{cp} } \right)_{nf} }}u^{2} = \alpha_{nf} \left( {\left| {u_{y} } \right|^{n - 1} T_{y} } \right)_{y} . $$

Individually, $\rho_{nf}$, $\mu_{nf}$, $\sigma_{nf}$ and $\alpha_{nf}$ are the density, viscosity, electrical conductivity and thermal diffusivity of nanofluid and is characterized as:

7 $$ \, \rho_{nf} = \left( {1 - \phi } \right)\rho_{f} + \phi \rho_{s} , \, \mu_{nf} = \frac{{\mu_{f} }}{{\left( {1 - \phi } \right)^{2.5} }},\alpha_{nf} = \frac{{k_{nf} }}{{\left( {\rho C_{p} } \right)_{nf} }}. $$

The volume fraction $\phi$, heat capacitance $k_{nf}$, and thermal conductivity $\left( {\rho C_{p} } \right)_{nf}$ are stated as

8 $$ \left( {\rho C_{p} } \right)_{nf} = \left( {1 - \phi } \right)\left( {\rho C_{p} } \right)_{f} + \phi \left( {\rho C_{p} } \right){}_{s},\,\frac{{k_{nf} }}{{k_{f} }} = \frac{{k_{s} + 2k_{f} - 2\phi \left( {k_{f} - k_{s} } \right)}}{{k_{s} + 2k_{f} + 2\phi \left( {k_{f} - k_{s} } \right)}}. $$

The Marangoni convection flow due to the surface tension gradient cannot be overlooked. Generally the surface tension $\sigma_{1}$ of the fluid film changes in a linear manner with respect to temperature described as $\sigma_{1} = \sigma_{0} \left[ {1 - \gamma \left( {T - T_{0} } \right)} \right]$. Further $\gamma = - \frac{1}{{\sigma_{0} }} \cdot \frac{{\partial \sigma_{1} }}{\partial t}$ shows the thermal coefficient^30^ while the shear stress in combination of surface tension along interface is given by $\tau = \frac{\partial \sigma }{{\partial x}}$^30^. Using operator $1 + \lambda \frac{D}{Dt}$ to the formula, according to Eq. (2), the modified Marangoni convection boundary condition is as follow:

9 $$ \sigma_{T} T_{x} + \lambda \sigma_{T} \left( {T_{tx} + uT_{xx} + vT_{ty} } \right) = \mu_{nf} \left| {u_{y} } \right|^{n - 1} u_{y} \,at\,\,\,y = h\left( {x,t} \right). $$

In Eq. (9) $h\left( {x,t} \right)$ represents the thickness of the uniform film:

10 $$ \begin{aligned} u = &U_{w} ,v = 0,T = T_{w} ,\,\,at\,y = 0; \hfill \\ v =& uh_{x} + h_{t} ,u_{y} = 0,T_{y} = 0,\,\,\,\,\,at\,\,\,y = h\left( {x,t} \right). \hfill \\ \end{aligned} $$

The group along with stream function is given by^8^

11 $$ \begin{aligned} \eta =& b^{{\frac{2 - n}{{n + 1}}}} v_{f}^{{ - \frac{1}{n + 1}}} x^{{\frac{1 - n}{{n + 1}}}} \left( {1 - \alpha t} \right)^{{\frac{n - 2}{{n + 1}}}} y,\psi = b^{{\frac{2n - 1}{{n + 1}}}} v_{f}^{{\frac{1}{n + 1}}} x^{{\frac{2n}{{n + 1}}}} \left( {1 - \alpha t} \right)^{{\frac{2n - 1}{{n + 1}}}} f\left( \eta \right),u = \psi_{y} ,v = - \psi_{x} \hfill \\ T =& T_{0} + T_{ref} dbx^{{\frac{3n + 1}{{n + 1}}}} \left( {1 - \alpha t} \right)^{{ - \frac{3n}{{n + 1}}}} \theta \left( \eta \right),\beta = h\left( {x,t} \right)b^{{\frac{2 - n}{{n + 1}}}} v_{f}^{{ - \frac{1}{n + 1}}} x^{{\frac{1 - n}{{n + 1}}}} \left( {1 - \alpha t} \right)^{{\frac{n - 2}{{n + 1}}}} . \hfill \\ \end{aligned} $$

By incorporating Eq. (11) into Eqs. (5) and (6) we have

12 $$ \begin{aligned} S\left( {f^{{\prime}{2}} - \eta \frac{n - 2}{{n + 1}}f^{\prime\prime}} \right) + & f^{{\prime}{2}} - \left( {\frac{2n}{{n + 1}}} \right)ff^{\prime\prime} + De\left[ {\frac{{4n^{2} }}{{\left( {n + 1} \right)^{2} }}f^{2} f^{\prime\prime\prime} - \frac{8n}{{\left( {n + 1} \right)^{2} }}ff^{\prime}f^{\prime\prime}} \right] \\ + & \frac{{\sigma_{f} }}{{\frac{{\rho_{nf} }}{{\rho_{f} }}}}Mf^{\prime} = \varepsilon_{1} \left( {\left| {f^{\prime\prime}} \right|^{n - 1} f^{\prime\prime}} \right)^{\prime } , \\ \end{aligned} $$

13 $$ S\left( {\frac{3n}{{n + 1}}\theta - \eta \left( {\frac{n - 2}{{n + 1}}} \right)\theta^{\prime}} \right) + \left[ {\left( {\frac{3n + 1}{{n + 1}}} \right)f^{\prime}\theta + \frac{1 - n}{{1 + n}}f\theta^{\prime}} \right] + MEc\theta f^{{\prime}{2}} = \frac{1}{pr}\varepsilon_{2} \left[ {\left| {f^{\prime\prime}} \right|^{n - 1} \theta^{\prime}} \right]^{\prime } . $$

While the related boundary conditions transformed to following form

14 $$ f^{\prime}\left( 0 \right) = 1,f\left( 0 \right) = 0,\theta \left( 0 \right) = 1,f\left( \beta \right) = \frac{2 - n}{{2n}}S\beta ,\theta^{\prime}\left( \beta \right) = 0. $$

Non-dimensionalize form of Marangoni convection boundary condition is as

15 $$ \left( {\left| {f^{\prime\prime}\left( \beta \right)} \right|} \right)^{n - 1} f^{\prime\prime}\left( \beta \right) = - M_{1} \left( {\frac{3n + 1}{{n + 1}}\theta \left( \beta \right) + D_{e} S\frac{3n}{{n + 1}}\frac{3n + 1}{{n + 1}}\theta \left( \beta \right) + D_{e} \frac{2n}{{n + 1}}\frac{3n + 1}{{n + 1}}f^{\prime}\left( \beta \right)\theta \left( \beta \right)} \right). $$

Above $\varepsilon_{1}$ and $\varepsilon_{2}$ are constants described in the resulting from^9^:

16 $$ \varepsilon_{1} = \frac{1}{{\left( {1 - \phi } \right)^{2.5} \left[ {\left( {1 - \phi } \right) + {{\phi \rho_{s} } \mathord{\left/ {\vphantom {{\phi \rho_{s} } {\rho_{f} }}} \right. \kern-\nulldelimiterspace} {\rho_{f} }}} \right]}},\varepsilon_{2} = \frac{{{{k_{nf} } \mathord{\left/ {\vphantom {{k_{nf} } {k_{f} }}} \right. \kern-\nulldelimiterspace} {k_{f} }}}}{{\left[ {\left( {1 - \phi } \right) + {{\phi \left( {\rho C_{p} } \right)_{s} } \mathord{\left/ {\vphantom {{\phi \left( {\rho C_{p} } \right)_{s} } {\left( {\rho C_{p} } \right)_{f} }}} \right. \kern-\nulldelimiterspace} {\left( {\rho C_{p} } \right)_{f} }}} \right]}}, $$

where $D_{e} = {{\lambda b} \mathord{\left/ {\vphantom {{\lambda b} {\left( {1 - \alpha t} \right)}}} \right. \kern-\nulldelimiterspace} {\left( {1 - \alpha t} \right)}}$ denotes the Deborah number concerned with relaxation time, $S = \frac{\alpha }{b}$ indicates the dimensionless unsteadiness parameter, $M{ = }{{\sigma_{f} \beta_{0}^{2} } \mathord{\left/ {\vphantom {{\sigma_{f} \beta_{0}^{2} } {\left( {\rho_{f} b} \right)}}} \right. \kern-\nulldelimiterspace} {\left( {\rho_{f} b} \right)}}$ denotes the magnetic factor, $\Pr =  \frac{\nu_{f}}{\alpha }$ means a Prandtl number, $M_{1} = \mu^{{ - \frac{1}{1 + n}}} \rho^{{ - \frac{1}{1 + n}}} b^{{\frac{1 - 2n}{{1 + n}}}} T_{ref} d\sigma_{0} \gamma$ represents the Marangoni number, $E_{c} = {{U_{w}^{2} } \mathord{\left/ {\vphantom {{U_{w}^{2} } {\left( {\left( {c_{p} } \right)_{nf} \left( {T_{s} - T_{0} } \right)} \right)}}} \right. \kern-\nulldelimiterspace} {\left( {\left( {c_{p} } \right)_{nf} \left( {T_{s} - T_{0} } \right)} \right)}}$ signify Eckert number.

## HAM solution

Semi analytical technique HAM is employed to solve the modeled problem in dimensionless form with the help of Mathematics Package-10. The detail of the solution is mentioned below:

17 $$ {{\hat{f}}_{0}} (\eta ) = \frac{{2n\eta^{3} - 2S\eta^{3} + nS\eta^{3} - 6n\eta^{2} \beta + 6S\eta^{2} \beta - 3nS\eta^{2} \beta + 4n\eta \beta^{2} }}{{4n\beta^{2} }}, \, {{\hat{\theta }}_{0}} (\eta ) = 1{ ,} $$

where the linear operators denoted by $L_{{\hat{f}}},\,{{\text{L}}_{{\hat{\theta }}}}$

18 $$ {L_{{\hat{f}}}} (\hat{f}) = {\hat{f}}^{\prime\prime\prime}, \,{{\text{ L}}_{{\hat{\theta }}}} {(}\hat{\theta }{) = }{{\hat{\theta }}{^{\prime\prime}}}{,} $$

where

19 $$ L_{{\hat{f}}} (e_{1} + e_{2} \eta + e_{3} \eta^{2} ) = 0,\,{\text{ L}}_{{\hat{\theta }}} (e_{4} + e_{5} \eta ) = 0{,} $$

where $e_{1} ,e_{2}$ and $e_{3}$ are constants.

The consistent non-linear operators $N_{{\hat{f}}} ,N_{{\hat{\theta }}} \,$ are prudently designated as:

20 $$ \begin{aligned}  N_{{\hat{f}}} {\mkern 1mu} \left[ {\hat{f}(\eta ;\zeta )} \right] = & S\left( {\hat{f}^{{\prime 2}} - \eta \frac{{n - 2}}{{n + 1}}\hat{f}^{{\prime \prime }} } \right) + \hat{f}^{{\prime 2}} - \left( {\frac{{2n}}{{n + 1}}} \right)\hat{f}\hat{f}^{{\prime \prime }} \\   & + De\left[ {\frac{{4n^{2} }}{{\left( {n + 1} \right)^{2} }}\hat{f}^{2} \hat{f}^{{\prime \prime \prime }} - \frac{{8n}}{{\left( {n + 1} \right)^{2} }}\hat{f}\hat{f}^{\prime } \hat{f}^{{\prime \prime }} } \right] + \frac{{\sigma _{f} }}{{\frac{{\rho _{{nf}} }}{{\rho _{f} }}}}M\hat{f}^{\prime } = \left( {\left| {\hat{f}^{{\prime \prime }} } \right|^{{n - 1}} \hat{f}^{{\prime \prime }} } \right)^{\prime } , \\ \end{aligned}  $$

21 $$ \begin{aligned} N_{{\hat{\theta }}} \left[ {\hat{f}(\eta ;\zeta ),\hat{\theta }(\eta ;\zeta )} \right] = & S\left( {\frac{3n}{{n + 1}}\hat{\theta } - \eta \left( {\frac{n - 2}{{n + 1}}} \right)\hat{\theta^{\prime}}} \right) \\ & + \left[ {\left( {\frac{3n + 1}{{n + 1}}} \right)\hat{f^{\prime}}\hat{\theta } + \frac{1 - n}{{1 + n}}\hat{f}\hat{\theta^{\prime}}} \right] + MEc\hat{\theta }\hat{f^{\prime}}^{2} = \frac{1}{pr}\varepsilon_{2} \left[ {\left| {\hat{f^{\prime\prime}}} \right|^{n - 1} \hat{\theta^{\prime}}} \right]^{\prime } . \\ \end{aligned} $$

For Eqs. (12) and (13) the 0th-order system as:

22 $$ \left( {1 - \zeta } \right)L_{{\overset{\lower0.5em\hbox{$\smash{\scriptscriptstyle\frown}$}}{f} }} \, \left[ {\overset{\lower0.5em\hbox{$\smash{\scriptscriptstyle\frown}$}}{f} (\eta ;\,\,\zeta ) - \overset{\lower0.5em\hbox{$\smash{\scriptscriptstyle\frown}$}}{f}_{0} (\eta )} \right] = P\hbar_{{\overset{\lower0.5em\hbox{$\smash{\scriptscriptstyle\frown}$}}{f} }} N_{{\overset{\lower0.5em\hbox{$\smash{\scriptscriptstyle\frown}$}}{f} }} \left[ {\overset{\lower0.5em\hbox{$\smash{\scriptscriptstyle\frown}$}}{f} (\eta ;\,\,\zeta )} \right], $$

23 $$ \left( {1 - \zeta } \right)L_{{\overset{\lower0.5em\hbox{$\smash{\scriptscriptstyle\frown}$}}{\theta } }} \, \left[ {\overset{\lower0.5em\hbox{$\smash{\scriptscriptstyle\frown}$}}{\theta } (\eta ;\,\,\zeta ) - \overset{\lower0.5em\hbox{$\smash{\scriptscriptstyle\frown}$}}{\theta }_{0} (\eta )} \right] = P\hbar_{{\overset{\lower0.5em\hbox{$\smash{\scriptscriptstyle\frown}$}}{\theta } }} N_{{\overset{\lower0.5em\hbox{$\smash{\scriptscriptstyle\frown}$}}{\theta } }} \left[ {\overset{\lower0.5em\hbox{$\smash{\scriptscriptstyle\frown}$}}{\theta } (\eta ;\,\,\zeta ),\overset{\lower0.5em\hbox{$\smash{\scriptscriptstyle\frown}$}}{f} (\eta ;\,\,\zeta )} \right]. $$

While BCs are:

24 $$ \begin{gathered} \left. {\frac{{\partial \overset{\lower0.5em\hbox{$\smash{\scriptscriptstyle\frown}$}}{f} (\eta ;\zeta )}}{\partial \eta }} \right|_{\eta = 0} = 0, \, \left. {\overset{\lower0.5em\hbox{$\smash{\scriptscriptstyle\frown}$}}{f} (\eta ;\zeta )} \right|_{\eta = 0} = 0,\left. {\overset{\lower0.5em\hbox{$\smash{\scriptscriptstyle\frown}$}}{\theta } (\eta ;\zeta )} \right|_{\eta = 0} = 0, \hfill \\ \left. {\overset{\lower0.5em\hbox{$\smash{\scriptscriptstyle\frown}$}}{f} (\eta ;\zeta )} \right|_{\eta = \beta } = \frac{2 - n}{{2n}}S\beta ,\left. {\frac{{\partial^{2} \overset{\lower0.5em\hbox{$\smash{\scriptscriptstyle\frown}$}}{f} (\eta ;\zeta )}}{{\partial \eta^{2} }}} \right|_{\eta = \beta } = 0,,\left. {\overset{\lower0.5em\hbox{$\smash{\scriptscriptstyle\frown}$}}{\theta } (\eta ;\zeta )} \right|_{\eta = \beta } = 0. \hfill \\ \end{gathered} $$

While the embedding constraint is $$\zeta \in [0,1]$$, to regulate for the solution convergence $$\hbar_{{\overset{\lower0.5em\hbox{$\smash{\scriptscriptstyle\frown}$}}{f} }}$$ and $$\hbar_{{\overset{\lower0.5em\hbox{$\smash{\scriptscriptstyle\frown}$}}{\theta } }}$$ are used. When $$\zeta = 0{\text{ and }}\zeta = 1$$ we have:

25 $$ \overset{\lower0.5em\hbox{$\smash{\scriptscriptstyle\frown}$}}{f} (\eta ;1) = \overset{\lower0.5em\hbox{$\smash{\scriptscriptstyle\frown}$}}{f} (\eta ),\overset{\lower0.5em\hbox{$\smash{\scriptscriptstyle\frown}$}}{\theta } (\eta ;1) = \overset{\lower0.5em\hbox{$\smash{\scriptscriptstyle\frown}$}}{\theta } (\eta ). $$

Expand the $$\overset{\lower0.5em\hbox{$\smash{\scriptscriptstyle\frown}$}}{f} (\eta ;\zeta ) \, $$ and $$\overset{\lower0.5em\hbox{$\smash{\scriptscriptstyle\frown}$}}{\theta } (\eta ;\zeta )$$ through Taylor’s series for $$\zeta = 0$$

26 $$ \begin{gathered} \overset{\lower0.5em\hbox{$\smash{\scriptscriptstyle\frown}$}}{f} (\eta ;\zeta ) \, = \, \overset{\lower0.5em\hbox{$\smash{\scriptscriptstyle\frown}$}}{f}_{0} (\eta ) + \sum\nolimits_{n = 1}^{\infty } {\overset{\lower0.5em\hbox{$\smash{\scriptscriptstyle\frown}$}}{f}_{n} (\eta )\zeta^{n} } \hfill \\ \overset{\lower0.5em\hbox{$\smash{\scriptscriptstyle\frown}$}}{\theta } (\eta ;\zeta ) \, = \, \overset{\lower0.5em\hbox{$\smash{\scriptscriptstyle\frown}$}}{\theta }_{0} (\eta ) + \sum\nolimits_{n = 1}^{\infty } {\overset{\lower0.5em\hbox{$\smash{\scriptscriptstyle\frown}$}}{\theta }_{n} (\eta )\zeta^{n} } , \hfill \\ \end{gathered} $$

28 $$ \overset{\lower0.5em\hbox{$\smash{\scriptscriptstyle\frown}$}}{f}_{n} (\eta ) \, = \left. {\frac{1}{n!}\frac{{\partial \overset{\lower0.5em\hbox{$\smash{\scriptscriptstyle\frown}$}}{f} (\eta ;\zeta )}}{\partial \eta }} \right|_{p = 0} ,\overset{\lower0.5em\hbox{$\smash{\scriptscriptstyle\frown}$}}{\theta }_{n} (\eta ) \, = \left. {\frac{1}{n!}\frac{{\partial \overset{\lower0.5em\hbox{$\smash{\scriptscriptstyle\frown}$}}{\theta } (\eta ;\zeta )}}{\partial \eta }} \right|_{p = 0} {. } $$

While BCs are:

29 $$ \begin{gathered} \overset{\lower0.5em\hbox{$\smash{\scriptscriptstyle\frown}$}}{f^{\prime}} \left( 0 \right) = 1,\overset{\lower0.5em\hbox{$\smash{\scriptscriptstyle\frown}$}}{f} \left( 0 \right) = 0,\overset{\lower0.5em\hbox{$\smash{\scriptscriptstyle\frown}$}}{\theta } \left( 0 \right) = 1, \hfill \\ \overset{\lower0.5em\hbox{$\smash{\scriptscriptstyle\frown}$}}{f} \left( \beta \right) = \frac{2 - n}{{2n}}S\beta ,\,\,\overset{\lower0.5em\hbox{$\smash{\scriptscriptstyle\frown}$}}{f^{\prime\prime}} \left( \beta \right) = 0,\overset{\lower0.5em\hbox{$\smash{\scriptscriptstyle\frown}$}}{\theta^{\prime}} \left( \beta \right) = 0. \hfill \\ \end{gathered} $$

Now

30 $$ \begin{aligned} \Re_{n}^{{\overset{\lower0.5em\hbox{$\smash{\scriptscriptstyle\frown}$}}{f} }} \left( \eta \right) = & S\left( {\overset{\lower0.5em\hbox{$\smash{\scriptscriptstyle\frown}$}}{f^{\prime}}_{n - 1}^{2} - \eta \frac{n - 2}{{n + 1}}\overset{\lower0.5em\hbox{$\smash{\scriptscriptstyle\frown}$}}{f^{\prime\prime}}_{n - 1} } \right) + \overset{\lower0.5em\hbox{$\smash{\scriptscriptstyle\frown}$}}{f^{\prime}}_{n - 1}^{2} - \left( {\frac{2n}{{n + 1}}} \right)\sum\limits_{j = 0}^{w - 1} {\overset{\lower0.5em\hbox{$\smash{\scriptscriptstyle\frown}$}}{f}_{w - 1 - j} \overset{\lower0.5em\hbox{$\smash{\scriptscriptstyle\frown}$}}{f^{\prime\prime}}_{j} } \\ & + De\left[ {\frac{{4n^{2} }}{{\left( {n + 1} \right)^{2} }}\sum\limits_{j = 0}^{w - 1} {\overset{\lower0.5em\hbox{$\smash{\scriptscriptstyle\frown}$}}{f}_{w - 1 - j}^{2} \overset{\lower0.5em\hbox{$\smash{\scriptscriptstyle\frown}$}}{f^{\prime\prime\prime}}_{j} } - \frac{8n}{{\left( {n + 1} \right)^{2} }}\sum\limits_{j = 0}^{w - 2} {\overset{\lower0.5em\hbox{$\smash{\scriptscriptstyle\frown}$}}{f}_{w - 2 - j} \overset{\lower0.5em\hbox{$\smash{\scriptscriptstyle\frown}$}}{f}_{w - 1 - j} \overset{\lower0.5em\hbox{$\smash{\scriptscriptstyle\frown}$}}{f^{\prime\prime}}_{j} } } \right] \\ & + \frac{{\sigma_{f} }}{{\frac{{\rho_{nf} }}{{\rho_{f} }}}}M\overset{\lower0.5em\hbox{$\smash{\scriptscriptstyle\frown}$}}{f^{\prime}}_{n - 1} - \varepsilon_{1} \left( {\sum\limits_{j = 0}^{w - 1} {\left| {\overset{\lower0.5em\hbox{$\smash{\scriptscriptstyle\frown}$}}{f^{\prime\prime}} } \right|_{w - 1 - j}^{n - 1} \overset{\lower0.5em\hbox{$\smash{\scriptscriptstyle\frown}$}}{f^{\prime\prime}}_{j} } } \right)^{\prime } , \\ \end{aligned} $$

31 $$ \begin{aligned} \Re_{n}^{{\overset{\lower0.5em\hbox{$\smash{\scriptscriptstyle\frown}$}}{\theta } }} (\eta ) =  S\left( {\frac{3n}{{n + 1}}\overset{\lower0.5em\hbox{$\smash{\scriptscriptstyle\frown}$}}{\theta }_{n - 1} - \eta \left( {\frac{n - 2}{{n + 1}}} \right)\overset{\lower0.5em\hbox{$\smash{\scriptscriptstyle\frown}$}}{\theta^{\prime}}_{n - 1} } \right) + \left[ {\left( {\frac{3n + 1}{{n + 1}}} \right)\sum\limits_{j = 0}^{w - 1} {\overset{\lower0.5em\hbox{$\smash{\scriptscriptstyle\frown}$}}{f^{\prime}}_{w - 1 - j} } \overset{\lower0.5em\hbox{$\smash{\scriptscriptstyle\frown}$}}{\theta }_{j} + \frac{1 - n}{{1 + n}}\sum\limits_{j = 0}^{w - 1} {\overset{\lower0.5em\hbox{$\smash{\scriptscriptstyle\frown}$}}{\theta }^{\prime}_{w - 1 - j} } \hat{f}_{j} } \right] \\ - MEc\sum\limits_{j = 0}^{w - 1} {\overset{\lower0.5em\hbox{$\smash{\scriptscriptstyle\frown}$}}{f^{\prime}}_{w - 1 - j}^{2} } \overset{\lower0.5em\hbox{$\smash{\scriptscriptstyle\frown}$}}{\theta }_{j} - \frac{1}{pr}\varepsilon_{2} \left[ {\sum\limits_{j = 0}^{w - 1} {\left| {\overset{\lower0.5em\hbox{$\smash{\scriptscriptstyle\frown}$}}{f^{\prime\prime}} } \right|_{w - 1 - j}^{n - 1} \overset{\lower0.5em\hbox{$\smash{\scriptscriptstyle\frown}$}}{\theta^{\prime}}_{j} } } \right]^{\prime } . \\ \end{aligned} $$

While

32 $$ \chi_{n} = \left\{ \begin{gathered} 0,{\text{ if }}n \le {1} \hfill \\ 1,{\text{ if }}n > {1}{\text{.}} \hfill \\ \end{gathered} \right. $$

## Results and discussion

**Figure 2:**
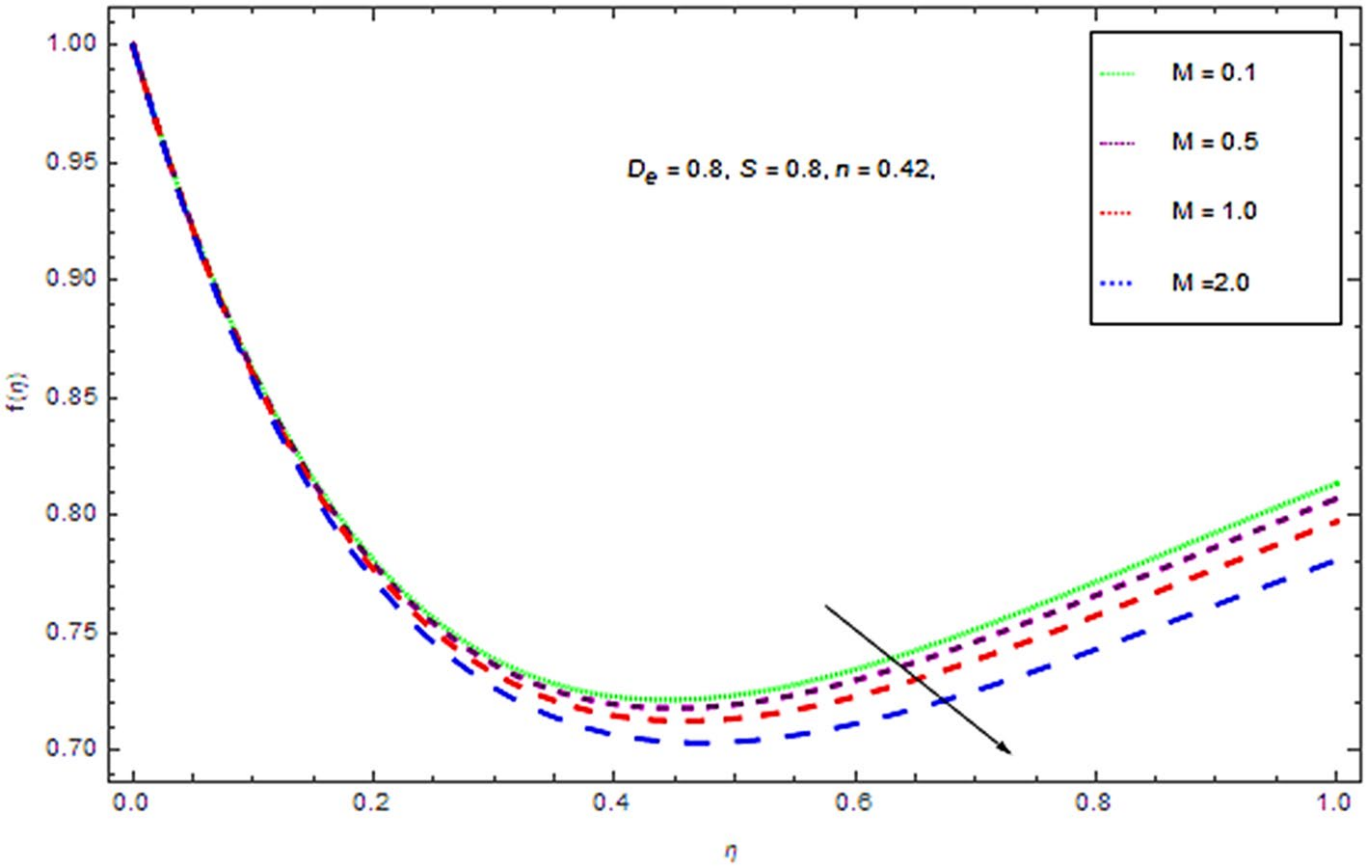
Influences of $M$ on $f(\eta )$.

**Figure 3:**
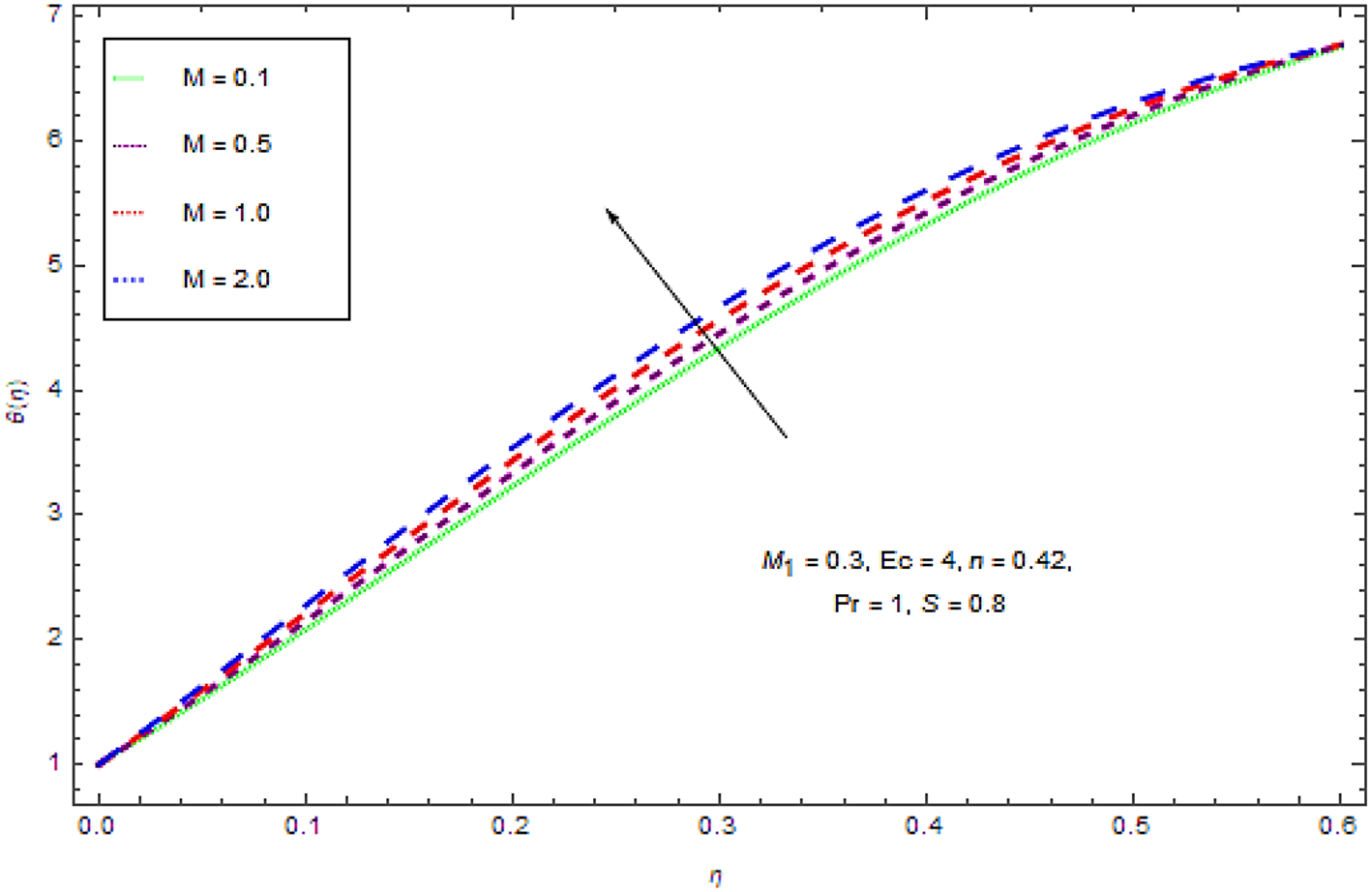
Effect of $M$ on $\theta (\eta )$.

**Figure 4:**
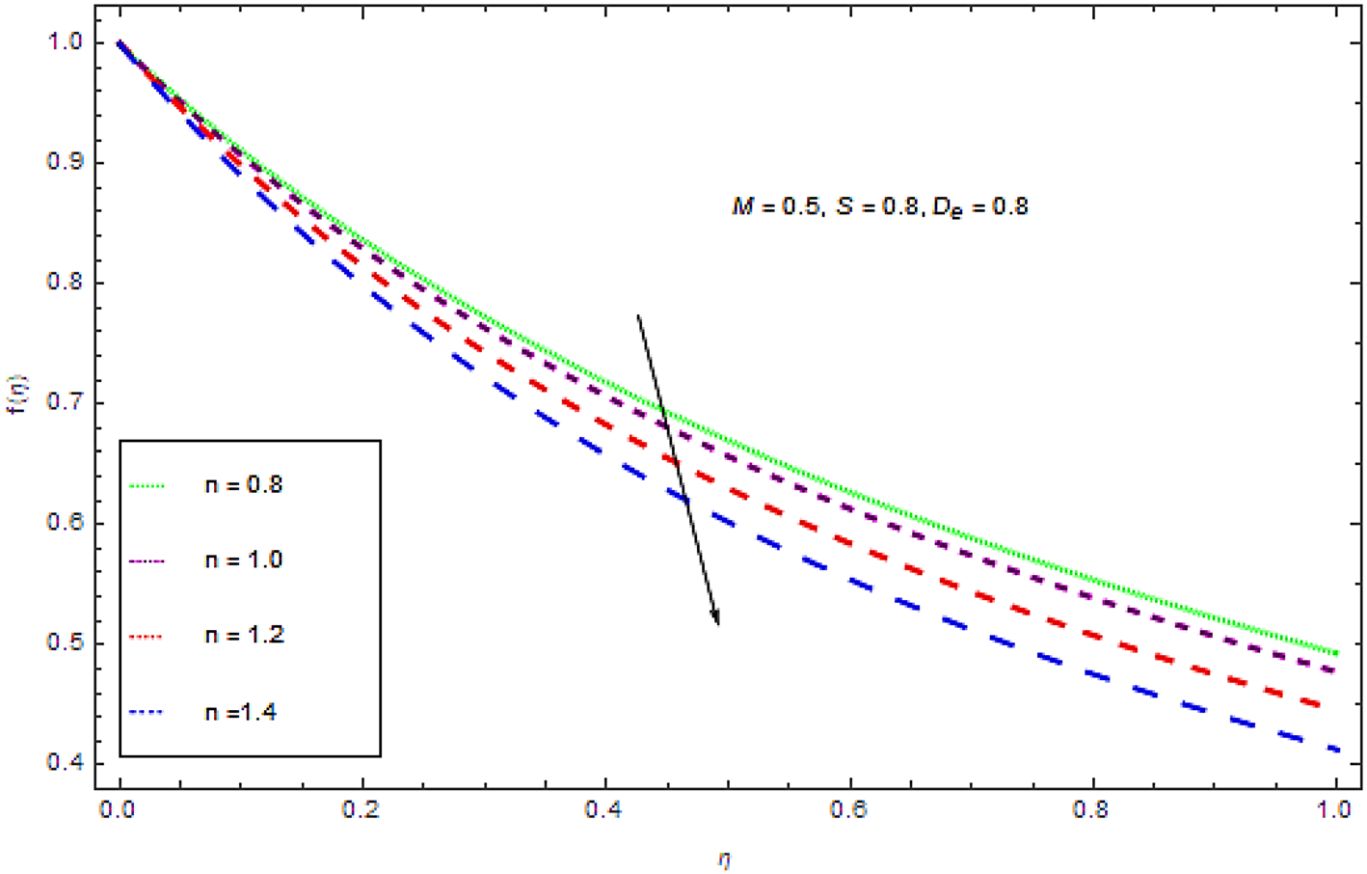
Impression of $n$ on $f(\eta )$.

**Figure 5:**
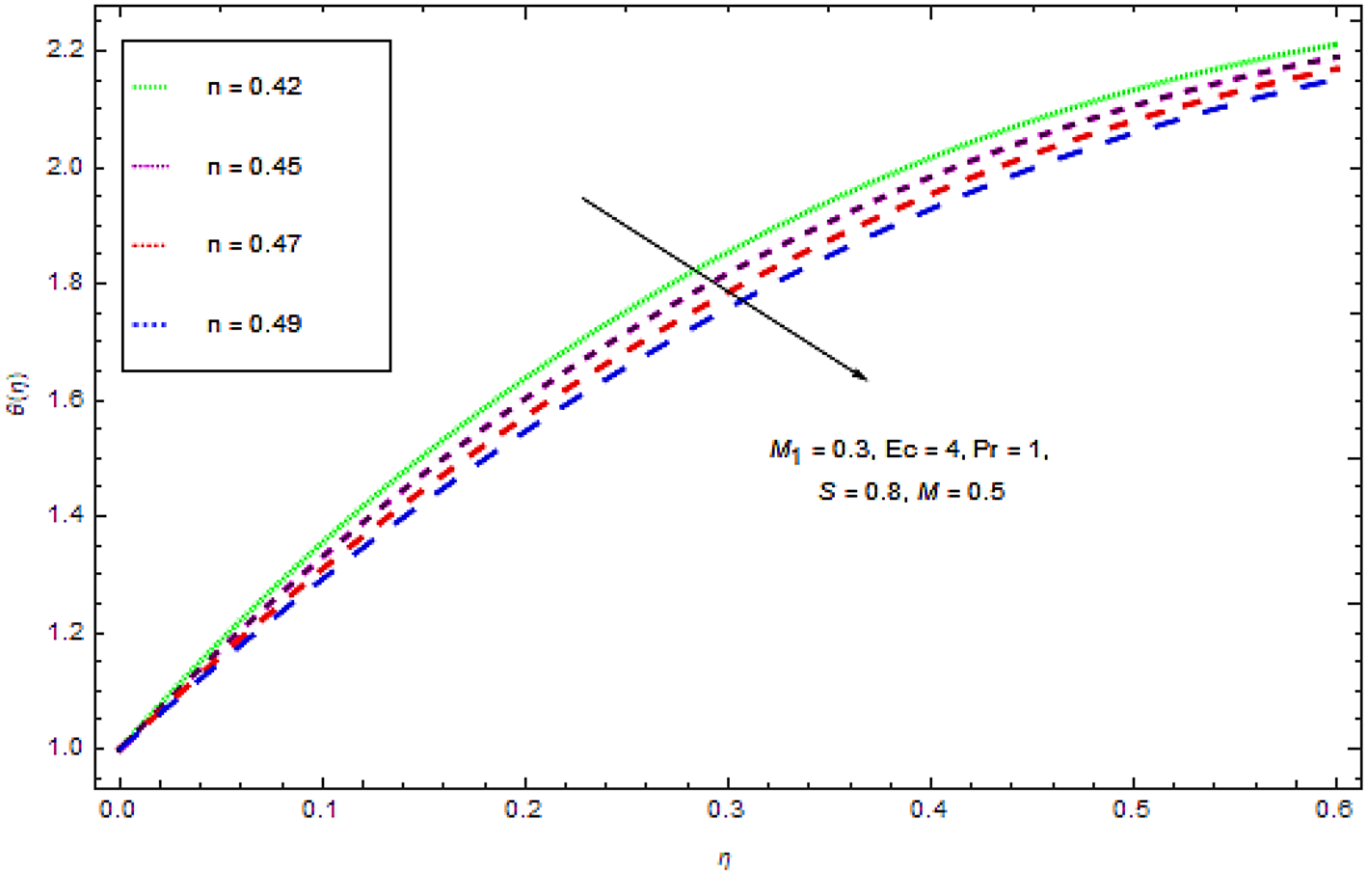
Effect of $n$ on $\theta (\eta )$.

**Figure 6:**
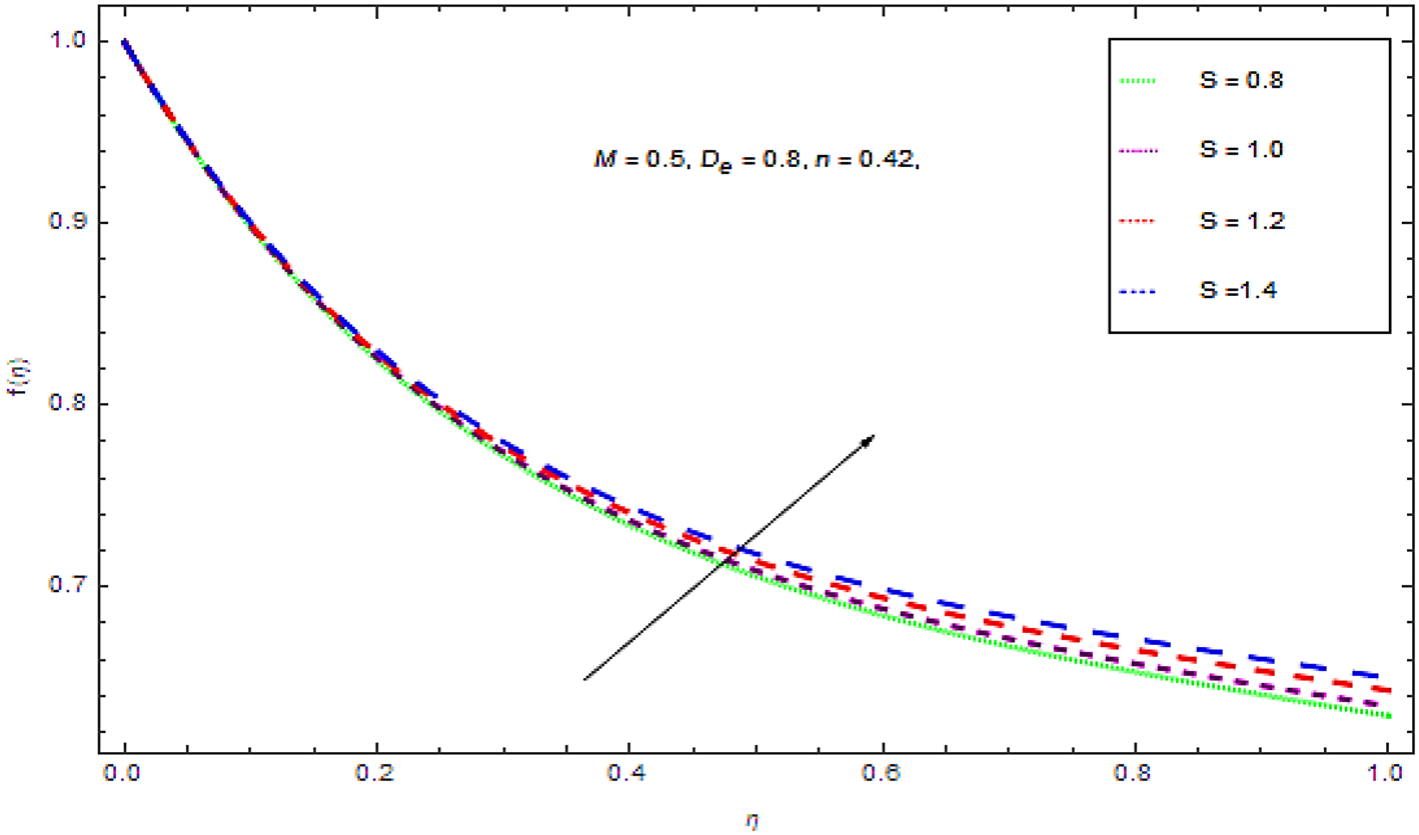
Outcome of $S$ on $f(\eta )$.

**Figure 7:**
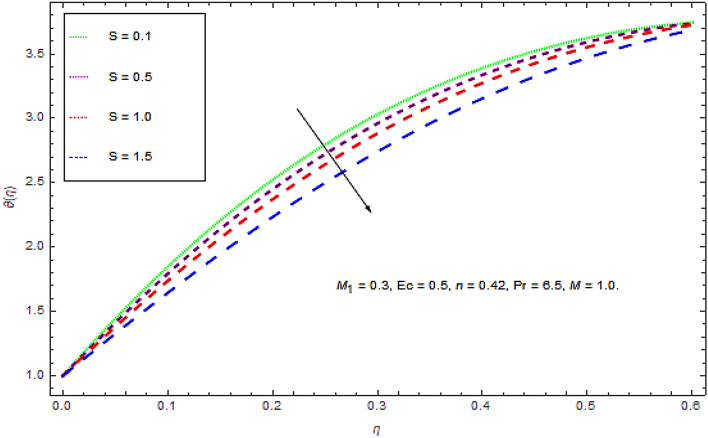
Impact of $S$ on $\theta (\eta )$.

**Figure 8:**
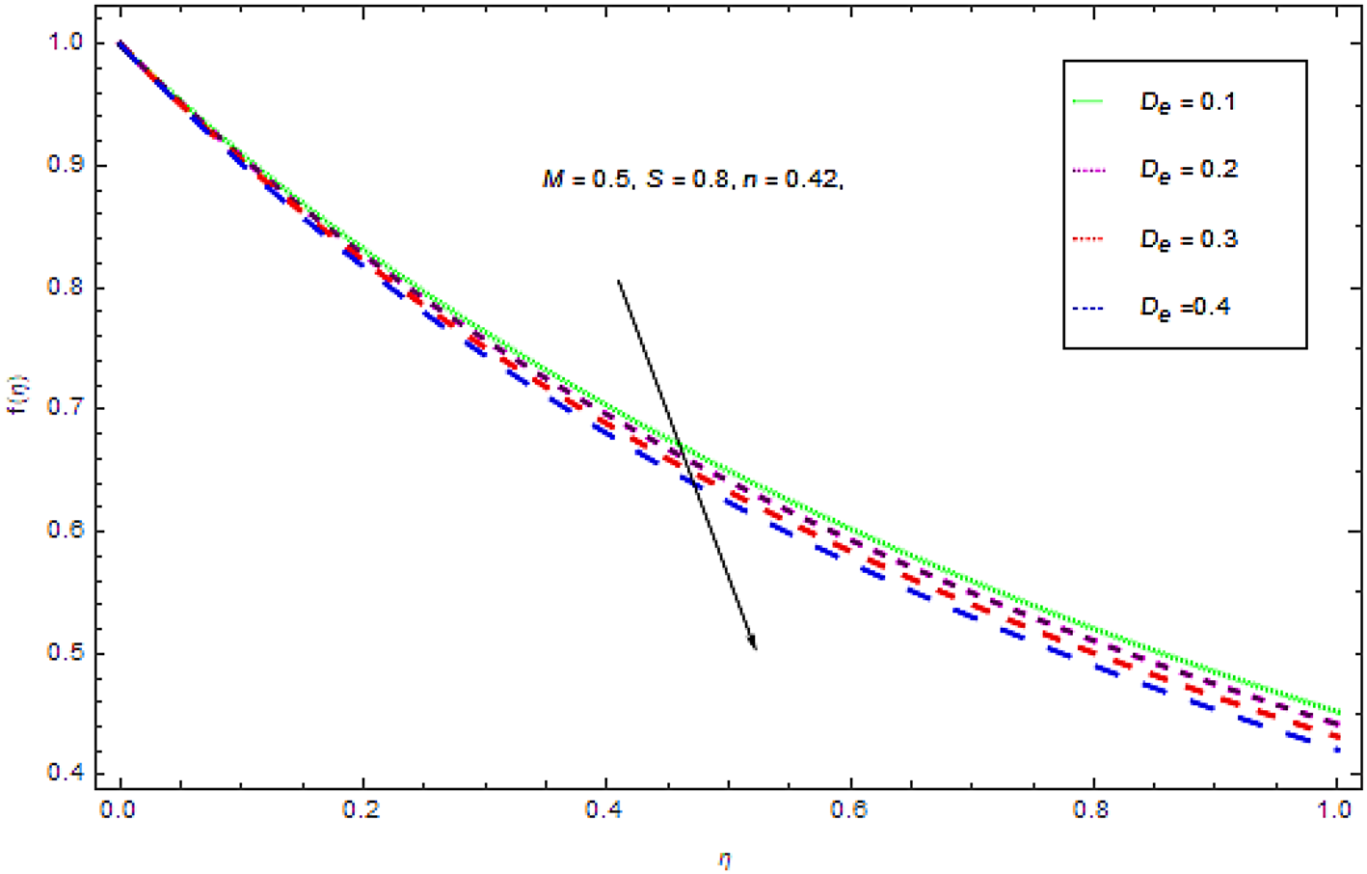
Influence of $D_{e}$ on $f(\eta )$.

**Figure 9:**
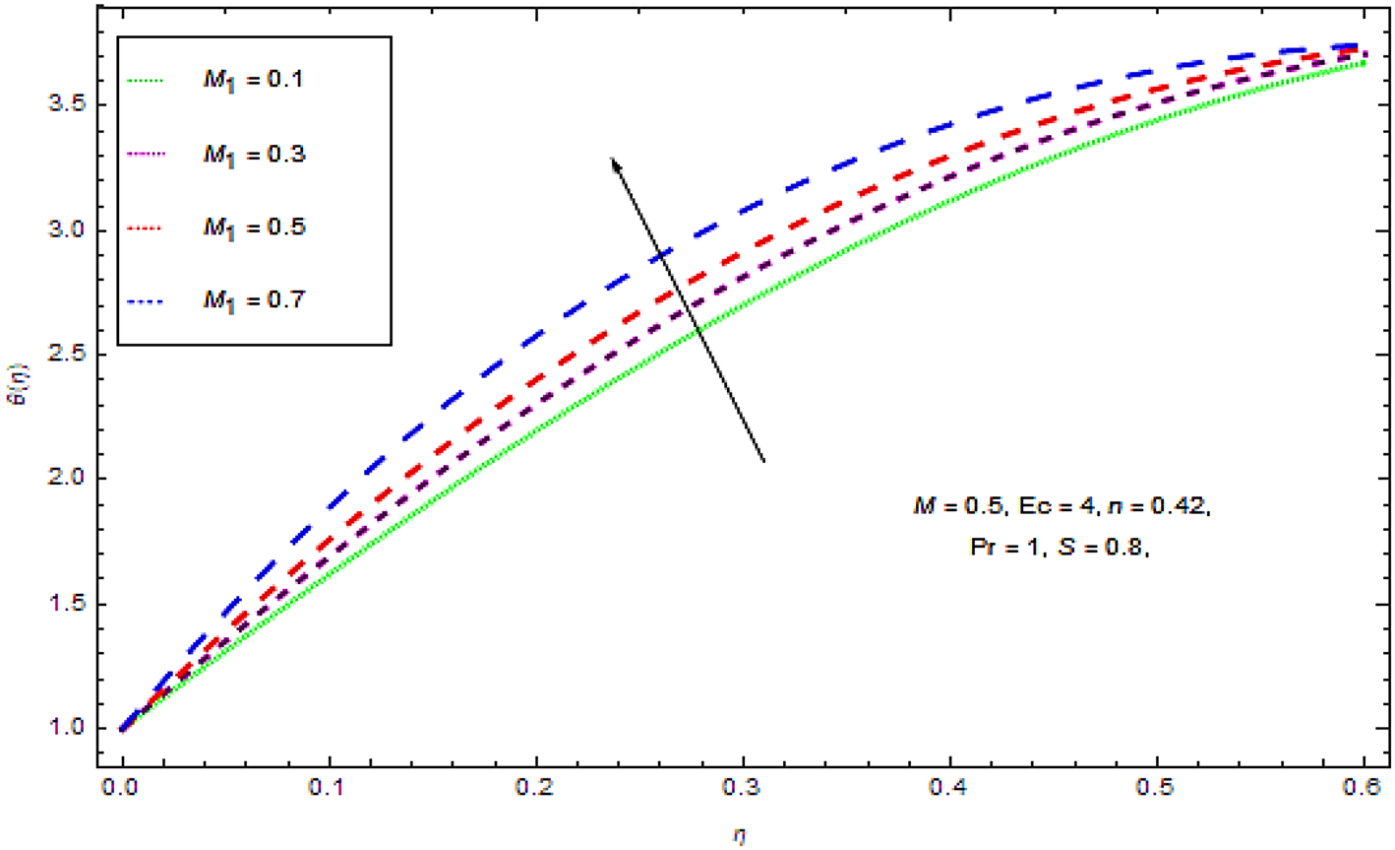
Effect of $M_{1}$ on $\theta (\eta )$.

**Figure 10:**
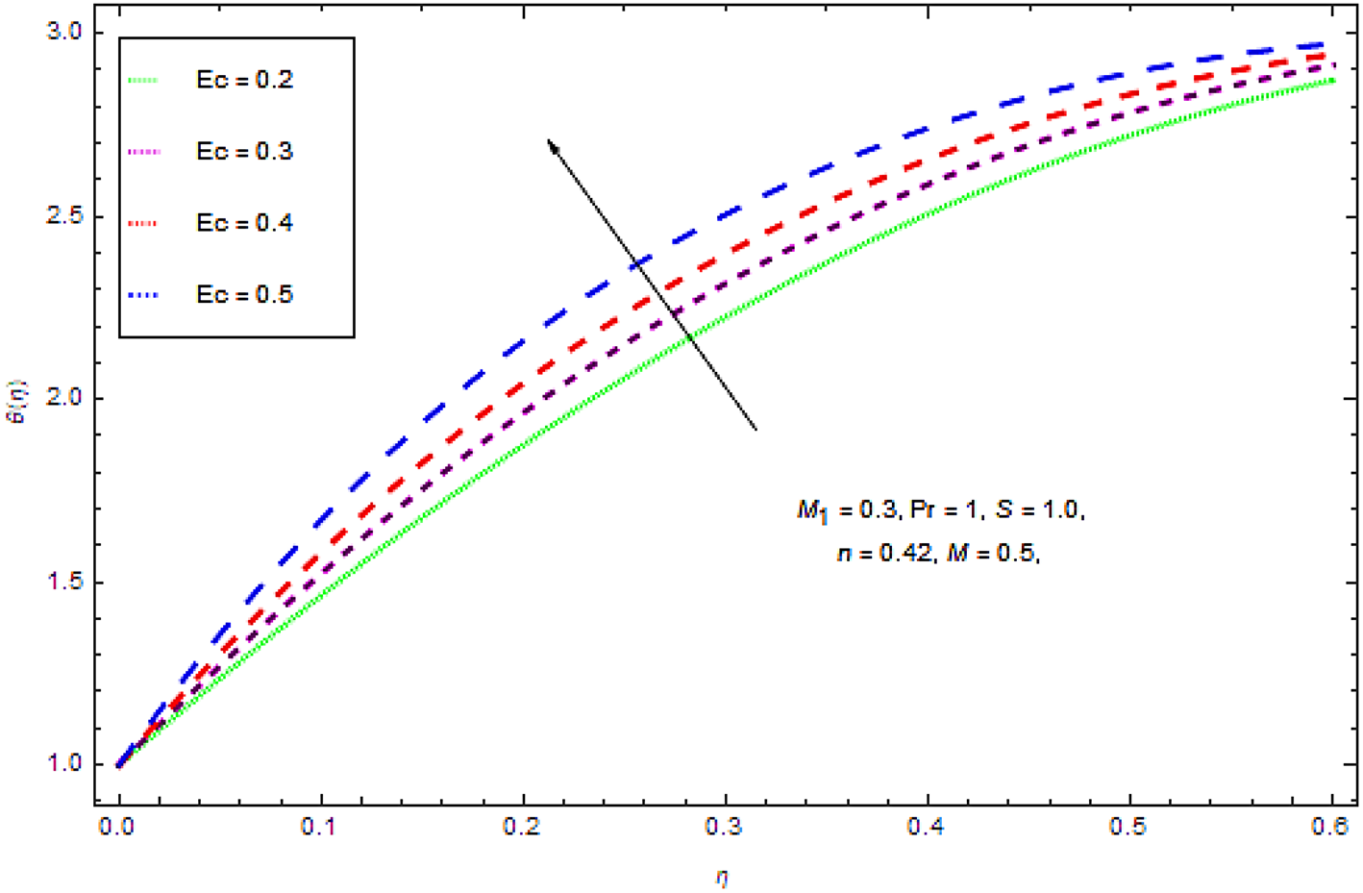
Outcome of $Ec$ on $f(\eta )$.

**Figure 11:**
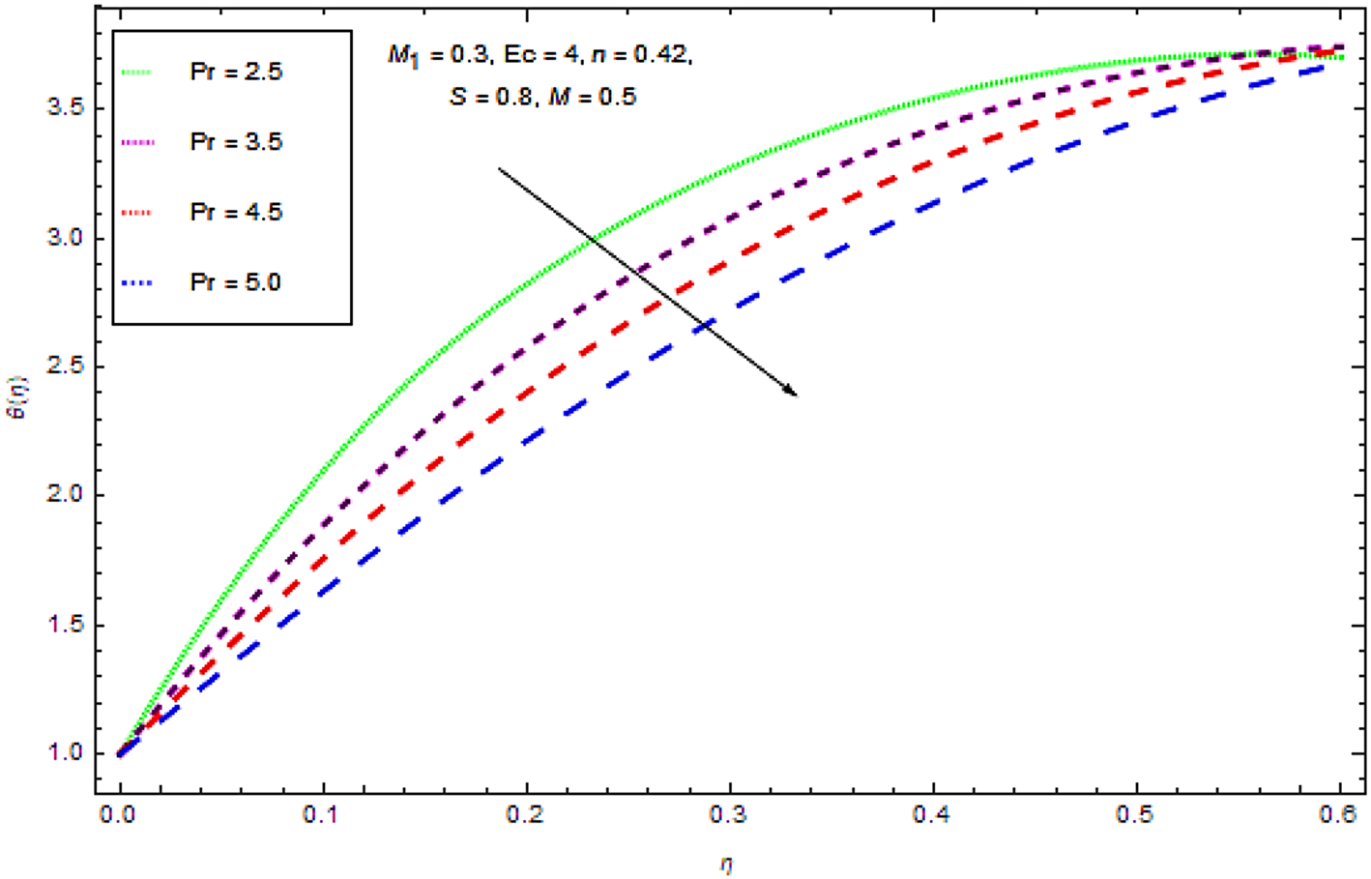
Effect of $\Pr$ on $\theta (\eta )$.

In the current investigation the modeled problem has transformed to dimensionless form by employing suitable set of similar variables. During this process some substantial parameters have been encountered that further have some influence upon flow system. In this section the impact of these emerging parameters upon flow system has discussed with the help of graphical view as presented in Figs. 2, 3, 4, 5, 6, 7, 8, 9, 10 and 11.

It appears from Figs. 2 and 3 that an upsurge in magnetic factor $M$ declines the velocity profiles for both Newtonian and non-Newtonian fluids and inverse for temperature profile. Actually the Lorentz force resists the flow and acts in the direction opposite to the flow direction. This resistive force slows down the fluid motion and increases the temperature of the fluid. Moreover, the induced magnetic field boundary layer thickness grows up with augmenting values *M.* Physically it can be interpreted as the both applied well as induced magnetic fields are in similar direction. So with augmentation in magnetic field there is a reduction in flow of fluid while a growth in boundary layer thickness.

From the Figs. 4 and 5, it is clear that flow and thermal characteristics decline with increasing values of power law index. These figures present that at a particular location $\eta$ the thermal as well as flow profiles decline with augmenting values of power law index that leads a decline in the thickness of boundary layer.

The impacts of unsteady constraint on the liquid velocity are established in Fig. 5. From this figure it is noticed that initially the flow along the surface declines with augmenting values of unsteadiness parameter that leads to a decreasing behavior in the momentum boundary layer thickness in the closed vicinity of the wall. On the other hand the flow of fluid grows up at locations away from the wall with augmenting values of unsteadiness parameter $S$. Hence the flow characteristics and boundary layer thickness seem to have reverse behavior for augmenting values of unsteady constraint.

Figure 7 determines the impact of unsteadiness parameter upon thermal characteristics. From this figure it is observed at some specific location the thermal characteristics seems to be declining with augmenting values of unsteadiness parameter $S$ that leads to a decrease in the heat transfer rate from fluid to the sheet. Moreover, it is worth mention that with higher values of $S$ the rate of cooling is much higher, while for steady flow the cooling may take longer time.

Figure 8 indicates that the augmenting values Deborah number $D_{e}$ leads a decline in the flow characteristics. Since $D_{e}$ is the ratio of characteristic time to scale time of deformation. So when $D_{e}$ grows up the time of relaxation augments that leads to slow regaining process. It is also to be noticed that $D_{e} = 0$ leads to a pure viscous fluid.

Figure 9 reveals that the larger Marangoni number $M_{1}$ leads to a thinner film. Moreover, the variation in the values of $M_{1}$ results an upsurge in the temperature of the film.

Figure 10 recognizes the effect of Eckert numbers $Ec$ upon temperature. It is seen that in view of progress in Eckert number there is an upsurge in thermal energy transportation of Maxwell power-law fluid. Actually indicates Joule heating impacts. So, upsurge in $Ec$ increases the temperature of nanofluids due to this physical phenomenon of heat transportation, heat dissipation decays, and heat advection grows up.

Actually, the ratio of kinematic viscosity with thermal diffusivity is known as prandtl number Pr, thus Fig. 11 tells us the temperature decreases with Pr increases due to a reduction in heat diffusion.

Table 1 shows the impact of different physical parameters over the velocity profile of the moving fluid. The selected parameters to analyze the flow behavior of the active fluid are power law index $n$, stress tensor $S$, The Deborah number $De$. From Table 1 we clearly noted that the moving speed of the fluid enhances by enlarging the values of stress tensor $S$ while the contras behavior of fluid motion is detected for enlarging the numerical estimations of power law index $n,\,\,De,$ and magnetic field parameter $M$. Table 2 shows the heat transfer rate of the moving liquid, we noted that the heat transfer enhances for boosting Eckert number $Ec$ magnetic parameter $M$ and Stress tensor $S$ while the inverse action is noted for temperature $\theta (\xi )$ when we increase the estimation of Prandtl number $\Pr$ and index of power law $n$. Also we can see that from Table 2 that the rate of heat transfer of the active fluid enhances for the enhancing the estimation of Eckert number $Ec$, magnetic factor $M$, and Stress tensor $S$ while the reverse action is noted for Prandtl number $\Pr$, power law index $n$. The comparison of the present work with the existing literature is displayed in Table 3.

**Table 1:** Impact of various emerging parameters upon flow profile $f(\xi )$.

$n$	$S$	$De$	$M$	$f(\xi )$
0.42	0.35	0.45	0.55	$0.6583333$
0.45				$0.6027777$
0.48				$0.5541666$
	0.8			$1.5047619$
	1.0			$1.8809523$
	1.2			$2.2571428$
		0.1		$0.6583231$
		0.5		$0.6273403$
		0.9		$0.5867353$
			0.1	$0.6583333$
			0.5	$0.6142370$
			0.9	$0.5586593$

**Table 2:** Impact of merging parameters over temperature profile $\theta (\xi )$.

$\Pr$	$Ec$	$M$	$n$	$S$	$\theta (\xi )$
10.1	0.3	0.5	4.5	1.2	$1.1341768$
10.5					$0.9268929$
10.9					$0.9048295$
	0.2				$1.4811337$
	0.3				$1.9546484$
	0.4				$2.3122837$
		0.1			$1.3301129$
		0.5			$1.3315893$
		0.9			$1.3330594$
			0.1		$1.1318499$
			0.5		$1.1003496$
			0.9		$1.0727030$
				0.8	$1.2821395$
				1.0	$1.3067434$
				1.2	$1.3315893$

**Table 3:** Comparison of the present results with Ref^32^ considering only common terms $n = 0.2,De = 0.2.$

$S$	$\beta$	$f^{\prime\prime}(0)\,\,[{\text{Present}}]$	$f^{\prime\prime}(0)$ Ref.^32^
0.4	0.3	0.8653872	0.8653763
0.5		0.8664883	0.8664752
0.6		0.8676322	0.8676213
	0.5	0.92310321	0.92312121
	0.7	1.014214321	1.014203512
	0.9	1.225329123	1.225328012

## Conclusions

The variable magnetic field impacts on flow of incompressible Maxwell-power-law liquid in a finite film over a widening sheet are deliberated in this exploration. The flow is also exposed to Joule heating and magnetic effects. The Marangoni convection equation is also proposed for current investigation in light of the constitutive equations for Maxwell power law model. After detail investigation of the current flow problem the following points are highlighted:

- For great estimations of $M$ the nanofluid films velocity distribution declines and inverse impact for temperature profile.
- The growing estimations of $\Pr$, upsurges the surface temperature, where converse effect is producing for $S$ that the surface temperature diminishes because of great values of $S$.
- The unsteady parameter can develop the fluid velocity.
- The variations of $M_{1}$ shows an increment in temperature of the film.
- It is observed in this study that, the thickness of boundary layer declines with growing values of Deborah number $D_{e}$ or diminishing power-law index $n$.
- An upsurge temperature distribution is detected for better estimations of Eckert number and vice versa.

## Data Availability

The data that support the findings of this study are available from the corresponding author upon reasonable request.

## Symbols

$$V = \left( {u,v} \right)$$: Velocity field
$$\left( {x,y} \right)$$: Cartesian coordinates
$T_{0} \,\,$: Temperatures of the slit
A: Rivlin Eriksen tensor
$M$: Magnetic field parameter
$$\Pr$$: Prandtl number
$${\text{D}}_{{\text{e}}}$$: Deborah number
$$M_{1}$$: Maragoni number
$\sigma_{0}$: Surface tension of the slit
$$\sigma_{1}$$: Surface tension
$\beta$: Dimensionless film thickness
$$\theta \,$$: Dimensionless temperature
$\mu_{nf}$: Dynamic viscosity of nanofluid
$\rho_{nf}$: Density of nanofluid
$\beta_{0}$: Applied magnetic field
$$\lambda$$: Relaxation time
$n$: Power law index
$T$: Temperature of the fluid
$T_{ref}$: Positive reference temperature
$$s$$: Extra tensor
$k_{nf}$: Thermal conductivity of nanofluid
$$Ec$$: Eckert number
$b$: Initial stretching sheet
$$\alpha ,d$$: Positive constant
$k_{0}$: Consistency thermal coefficient
S: Unsteady parameter
$h\left( {x,t} \right)$: Uniform thin film thickness
$\alpha_{nf}$: Thermal diffusivity of nanofluid
$$\dot{\gamma }$$: Temperature coefficient
$$f^{\prime}$$: Dimensionless velocity
$\upsilon_{nf}$: Kinematic viscosity of nanofluid
$$\eta$$: Similarity variable
$^{\prime}$: Derivative with respect to $\eta$
$\sigma_{nf}$: Electrical conductivity of nanofluid
